# An Index Combining Lost and Remaining Nerve Fibers Correlates with Pain Hypersensitivity in Mice

**DOI:** 10.3390/cells9112414

**Published:** 2020-11-04

**Authors:** Han-Hsiung Chi, Jye-Chang Lee, Chih-Cheng Chen, Shih-Kuo Chen, Chen-Tung Yen

**Affiliations:** 1 Department of Life Science, National Taiwan University, Taipei City 10617, Taiwan; eric@zlabs.com.tw (H.-H.C.); alenskchen@ntu.edu.tw (S.-K.C.); 2 Molecular Imaging Center, National Taiwan University, Taipei City 10051, Taiwan; 2jclee@gmail.com; 3 Institute of Biomedical Sciences, Academia Sinica, Taipei City 11529, Taiwan; chih@ibms.sinica.edu.tw

**Keywords:** chronic constriction injury, cutaneous nerve fiber, intravital two-photon fluorescence microscopy, Nav1.8, neuropathic pain

## Abstract

Multiple peripheral nerves are known to degenerate after nerve compression injury but the correlation between the extent of nerve alteration and pain severity remains unclear. Here, we used intravital two-photon fluorescence microscopy to longitudinally observe changes in cutaneous fibers in the hind paw of Nav1.8-Cre-tdTomato mice after chronic constriction injury (CCI). Results showed that the CCI led to variable loss of the skin nerve plexus and intraepidermal nerve fibers. The timing of Nav1.8 nerve fiber loss correlated with the development of mechanical hypersensitivity. We compared a scoring approach that assessed whole-paw nerve degeneration with an index that quantified changes in the nerve plexus and terminals in multiple small regions of interest (ROI) from intravital images of the third and fifth toe tips. We found that the number of surviving nerve fibers was not linearly correlated with mechanical hypersensitivity. On the contrary, at 14 days after CCI, the moderately injured mice showed greater mechanical hypersensitivity than the mildly or severely injured mice. This indicates that both surviving and injured nerves are required for evoked neuropathic pain. In addition, these two methods may have the estimative effect as diagnostic and prognostic biomarkers for the assessment of neuropathic pain.

## 1. Introduction

Peripheral neuropathic pain is chronic pain caused by nerve injury and is characterized by allodynia, hyperalgesia and spontaneous pain. Nerve injury can result from disease, trauma and surgical injuries, nerve entrapment or drug toxicity. Although chronic neuropathic pain is common, no satisfactory treatment is available. Currently, intraepidermal fiber density (IEFD), which refers to the density of immunohistochemically-defined small fibers in a skin biopsy, is the gold standard for the clinical diagnosis of neuropathy. However, no correlation has been found between IEFD and neuropathic pain [1,2,3]. We hypothesize that an index combining the severity of nerve injury and the number of surviving nerves could be a useful intravital microscopic index for the diagnosis and prognosis of evoked neuropathic pain.

Two-photon fluorescence microscopy is an advanced technique that considerably reduces the undesirable effects of light scattering in thick tissues and ensures high axial resolution with low phototoxicity [4]. These advantages make intravital microscopy particularly suitable for observing and measuring living tissues such as the skin. Using transgenic mice that express fluorescent reporters in small-diameter nerve fibers, we performed longitudinal imaging of cutaneous nerve endings in their paws and correlated the resulting nerve scores with the initiation and maintenance of neuropathic pain.

We used the chronic constriction injury (CCI) model in the present study. CCI is a well-established animal model of entrapment nerve injury. CCI has been reported to induce both mechanical hypersensitivity and thermal hyperalgesia in rodents [5,6,7]. Moreover, the resulting increase in paw withdrawal frequency has been found to be sustained for more than 70 days after CCI surgery in mice [6]. Varying the tightness of constriction is one means of manipulating the extent of nerve injury [8]. This would enable scoring of the degree of nerve survival in the mice and this could then be correlated with their mechanical hypersensitivity.

Nociceptors are the primary transducers of noxious and/or potentially damaging stimuli from the periphery. The Nav1.8 channel is a subtype of the voltage-gated sodium channels that are present in most populations of nociceptors and pruriceptors [9] and they play a crucial role in the generation and propagation of action potentials. In the epidermis, Nav1.8-positive nerve fibers and free nerve endings colocalize with fibers that express PGP9.5, which is a general neuronal marker [10,11,12]. In addition, Nav1.8-expressing neurons are among the major players in pain onset and hypersensitivity in chronic pain [13]. The knockdown of Nav1.8 mRNA has been shown to reverse neuropathic pain behavior in the CCI model [14,15,16]. Furthermore, redistribution of the voltage-gated sodium channels Nav1.6, Nav1.8 and Nav1.9 contributed to the resolution of neuropathic pain in CCI-treated rats after nerve decompression [7]. In summary, the aforementioned studies strongly suggest that Nav1.8 is involved in CCI-induced neuropathic pain. Although peripheral nerve degeneration after entrapment nerve injury has been well established, the correlation between nerve alteration and pain severity remains unclear. The aim of this study was to determine whether nerve degeneration and nerve surviving may contribute to the evoked hypersensitivity. In this study, we used intravital fluorescence microscopy to longitudinally score changes in cutaneous fibers in the hind paw of a Nav1.8-knock-in mouse (Nav1.8-Cre-tdTomato) after CCI surgery. We developed a nerve-scoring approach to assess the level of whole-paw fiber degeneration at different timepoints following CCI surgery and correlated this score with CCI-induced mechanical hypersensitivity. We also used the product of nerve fiber score (surviving nerves) and nerve fibers lost (nerve injury) as an index for assessing the correlation between nerve damage and evoked mechanical hypersensitivity. Finally, we derived an index of changes in the nerve plexus and terminals in the third and fifth toe tips, which were quantified from multiple small regions of interest (ROI) in intravital images. We then compared the correlations between these indices and mechanical hypersensitivity and assessed their estimative effect for assessing neuropathic pain. Classical ATF-3 immunostaining was used as a marker of nerve injury for comparison [17,18,19].

## 2. Materials and Methods

### 2.1. Animals

The Nav1.8-Cre mice used in this study were obtained from Dr. John N. Wood [20] and crossed with tdTomato protein reporter mice to generate Nav1.8-CretdTomato mice. All mice were backcrossed with wild-type (WT) C57BL/6JNarl mice that were purchased from the National Laboratory Animal Center (Taipei, Taiwan). All mice were housed under a 12 h:12 h light-dark cycle at 22 °C and given food and water ad libitum before experiments. Adult mice (> 8 weeks old) of both sexes were used for experiments. All experimental and animal handling procedures were approved by The Institutional Animal Care and Use Committee of National Taiwan University (approval number: NTU105-EL-00113), in accordance with the relevant sections of the Animal Research: Reporting of In Vivo Experiments (ARRIVE) guidelines. The methods used in this study were compliant with the Codes for the Experimental Use of Animals of the Council of Agriculture of Taiwan, which is based on the Animal Protection Law of Taiwan.

### 2.2. Experimental Design

Wild-type (male, *n* = 11; female, *n* = 8) and Nav1.8-tdTomato mice (male, *n* = 13; female, *n* = 10) were used to assess CCI-induced neuropathic pain behavior. Group-housed mice (4–5 per cage) were randomly divided into a sham group and a CCI group for each experimental trial (WT-sham group, *n* = 7; WT-CCI group, *n* = 12; Nav-sham group, *n* = 8; Nav-CCI group, *n* = 15).

The CCI model was used to investigate the relationship between nerve injury and pain scores. CCI produces variable degrees of nerve injury that can be adjusted by manipulating the tightness of the compression [8]. Furthermore, the pain behavior resulting from CCI develops relatively slowly, which provides a useful time window for prognosis tests. Nav1.8-tdTomato mice were used for the longitudinal investigation of nerve condition in the hind paw, either globally (i.e., the whole paw; male, *n* = 27; female, *n* = 10) or in a small specific area (i.e., the toe tip; male, *n* = 14). Behavioral tests and intravital images were performed at baseline and on days 3, 7 and 14 after CCI surgery. To compare the diagnostic ability of classical ATF-3 staining with the intravital nerve-scoring approach, mice were decapitated three days after the final intravital imaging session.

### 2.3. Chronic Constriction Injury

The CCI model has been described previously [21,22]. Briefly, mice were anesthetized with an intraperitoneal injection of a ketamine (50 mg/kg) and xylazine (15 mg/kg) mixture. The left sciatic nerve was exposed at the mid-thigh level and loosely constricted using three ligatures of 6-0 silk sutures, with approximately 1 mm spacing between ligatures. The skin incision was closed using 4-0 silk sutures.

### 2.4. Nociceptive Tests for Pain Threshold Measurement

#### 2.4.1. Assessment of Mechanical Hypersensitivity

Mechanical hypersensitivity was evaluated in terms of the withdrawal response of the hind paw to von Frey stimulation, using the up-and-down method [23]. All tests were conducted during the day (09:00–18:00). Each mouse was placed in a transparent acrylic chamber (11.5 × 4.5 × 5 cm^3^) with a mesh floor for 3 d to ensure habituation before baseline testing. On the test day, after habituation for 20–30 min, a series of von Frey hairs (0.02, 0.04, 0.07, 0.16, 0.4, 0.6, 1.0 and 1.4 g; Touch Test Sensory Evaluator, North Coast Medical, Morgan Hill, CA, USA) were perpendicularly applied to the middle plantar surface of the hind paw. Stimulation commenced with the 0.16 g monofilament and lasted 5–8 s each. Clear paw withdrawal or shaking behavior during von Frey stimulation was defined as a positive response. Whenever a positive response occurred, the next lighter hair was used for stimulation and vice versa. The testing consisted of four stimuli after the first change in response occurred. Three repetitions were performed, with 1 min intervals between repetitions. The pattern of response was converted into the 50% withdrawal threshold.

#### 2.4.2. Assessment of Thermal Hyperalgesia

To obtain the nociceptive threshold of an acute thermal stimulus, paw withdrawal latencies were calculated using a radiant heat test (Planter test Model 390, IITC Life Science, Woodland Hills, CA, USA) as previously described [24]. The mice were placed in the same acrylic chamber as described above, which was mounted on top of a heated glass surface (Model 400 Heated Base, IITC Life Science, Woodland Hills, CA, USA). Mice were allowed to acclimate for 20 min before testing. A mobile radiant heat source located under the glass was focused on the plantar surface of the hind paw. The baseline values of paw withdrawal latencies were determined from the average of three responses. A minimum interval of 10 min was allowed between trials to prevent scalding and a cutoff time of 15 s per trial was used to prevent tissue damage. The intensity of the heat stimulus was kept constant throughout the experiment.

### 2.5. Immunohistochemistry

Mice were anesthetized using isoflurane and decapitated 10 d after CCI surgery. Bilateral lumbar dorsal root ganglions (DRG; L3–5) were dissected and postfixed in 4% paraformaldehyde (Merck, Darmstadt, Germany) for 30 min. The tissues were transferred to 30% glucose in phosphate-buffered saline (PBS) and stored at 4 °C overnight. They were then embedded in optimal cutting temperature (OCT) compound, rapidly frozen using dry ice and stored at −80 °C. For staining, 25 μm-thick DRG cryosections were cut, washed three to five times with PBS containing 0.3% Triton X-100 (PBST) and then blocked in 5% normal goat serum (NGS) (Vector Laboratories, Burlingame, CA, USA) for 1.5 h at room temperature. The primary antibody, rabbit anti-ATF3 (1:500, Sigma, St. Louis, MO, USA), was diluted in 0.3% PBST containing 1% NGS and used to treat the sections overnight at 4 °C. The secondary antibody, Alexa Fluor 488 goat anti-rabbit (1:1000; Jackson Immunoresearch, West Grove, PA, USA), was likewise diluted in 0.3% PBST containing 1% NGS and used to stain the sections for 2 h at room temperature. The sections were washed three to five times with 0.3% PBST and then mounted with RapiClear CS mounting solution (Sunjin Lab, Taipei, Taiwan). Sections were viewed using Leica TCS SP8 (Leica, Wetzlar, Germany) and processed with ImageJ Fiji [25]. The number of DRG neurons were calculated from at least four randomly selected 200 × 200 μm square areas from one image of an intact DRG section.

### 2.6. Intravital Microscopy

#### 2.6.1. Global Imaging Using Fluorescence Microscopy

Mice were anesthetized via intraperitoneal injection of a ketamine (50 mg/kg) and xylazine (15 mg/kg) cocktail. After washing the left hind paw with double-deionized (dd) water and 100% EtOH, the mice were positioned in an acrylic chamber (39.6 × 23.8 × 1.3 mm), fixed with Blu-tack clay (Bostik Australia Pty Ltd., Thomastown, VIC, Australia) and placed on a Zeiss observer Z1 fluorescence microscope (Carl Zeiss, Oberkochen, Germany) equipped with excitation (531/40 nm) and emission filters (593/40 nm). Images of the left hind paw were captured and processed using ImageJ Fiji.

**Figure 1:**
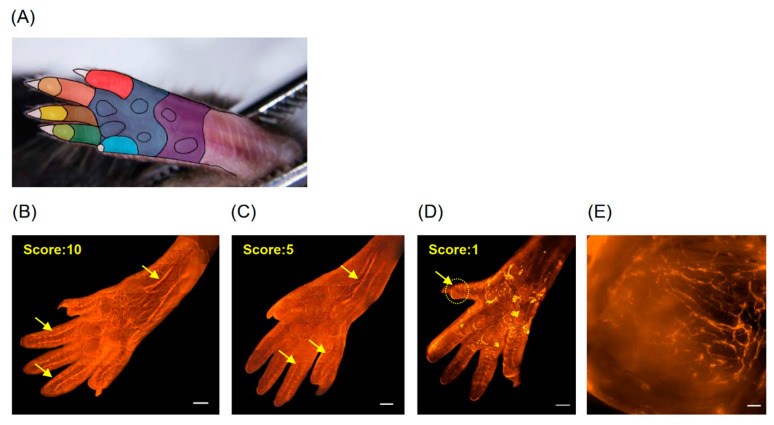
Scoring of chronic constriction injury (CCI)-induced nerve injury in the hind paw. Nav1.8-tdTomato nerve fibers were observed using a fluorescent microscope. (**A**) The underside of the hind paw was divided into 10 regions (eight in the five toes, two in the palm of the paw), as indicated by different colors. One point was scored for each area that exhibited a high degree of innervation. Examples of (**B**) a healthy hind paw scoring 10 points; (**C**) a moderately injured hind paw scoring 5 points; (**D**) a severely injured hind paw scoring only 1 point. Arrows indicate nerve fibers. (**E**) A higher-magnification view showing the nerve fibers remaining in the regions of interest (ROI) indicated in (**D**). Scale bars: 1000 μm (**B**–**D**) or 50 μm (**E**).

A 10-point scoring system was used to assess changes in the nerve terminals in the left hind paw after CCI-induced injury. The area of the hind paw was divided into 10 regions (Figure 1A). One point was scored for each region that exhibited a high degree of innervation. We found that CCI caused local sciatic nerve injury during its pathological development, which resulted in different patterns of nerve scoring (Figure 1B–D). Figure 1E is a higher-magnification view of the ROI from Figure 1D that shows the remaining nerve fibers in mice with severe CCI injury (CCI-severe) at 7 d after surgery (Figure 1E).

#### 2.6.2. Local Imaging Using Two-Photon Fluorescence Microscopy

Mice were anesthetized using 5% isoflurane and maintained under anesthesia with 1.5–2% isoflurane. The left hind paw was washed with dd water and 100% EtOH, positioned in a homemade acrylic chamber as described above for global imaging, fixed with Blu-tack clay and placed on a Leica TCS SP5 multiphoton microscope (Leica) equipped with a femtosecond-pulsed Ti:Sapphire laser (Chameleon Vision II; Coherent, Santa Clara, CA, USA). The hind paw was immersed in glycerol to match the refractive index. A 20× water-immersion objective was used. The excitation laser wavelength was set to approximately 1040 nm for tdTomato. The laser output power was adjusted by placing the power meter after the objective lens as a reference and it was set to 40–50 mW. Two individual photomultiplier tubes (PMTs) were set at 515–524 nm and 550–640 nm to detect second-harmonic generation signals and fluorescent signals, respectively, in tdTomato-positive mice. Images of the tips of the third and fifth toes were captured and analyzed using Amira software (Mercury Computer Systems/3D Viz group, San Diego, CA, USA).

#### 2.6.3. D Intravital Image Processing and Analysis

All 3D reconstruction and analysis of the intravital imaging was performed using Amira software (Mercury Computer Systems/3D Viz group). The 3D structures of the intravital images from the same digit at different observation times were reconstructed individually. All 3D-reconstructed images were registered with reference to matching landmarks in the fluorescent signal from the nerve plexus. After the realignment and co-registration of images had been performed, the largest possible overlapping volume in the images from different days was cropped. A baseline image was used as a template to retransform the cropped images. The quantification of nerve plexus and terminals was based on at least three sub-volumes. A non-local mean filter was used to remove noise from the input image while preserving the sharpness of strong edges. An interactive thresholding module was then used to binarize the image. The nerve plexus was further skeletonized and the total length of the nerve plexus and number of nerve terminals were calculated for each day. Nerve terminals that were not attached to the main plexus were ruled out from quantification and further analysis.

### 2.7. Statistical Analysis

GraphPad Prism 8.0.2 (GraphPad Software, San Diego, CA, USA) was used for statistical analyses and graphing. Data are presented as mean ± standard error of the mean (SEM). The level of statistical significance was set at 5% (*p* < 0.05) for all analyses. A two-way repeated measures analysis of variance (ANOVA) with Sidak multiple comparisons was used to analyze the nociceptive threshold in the behavioral results, with group and time since CCI surgery as factors. Linear regression analysis was used to examine the correlation between the nerve-scoring test results and the paw withdrawal threshold. To analyze the CCI subgroups, both one-way and two-way ANOVAs with Tukey’s multiple comparisons were used to compare the effects between the different toe tips and severity levels of the CCI surgery. The minimum total sample size (*n* = 36) was calculated using G*Power 3.1.9.6 software [26]. The effect size, α-level and power (0.25, 0.05 and 0.8, respectively) were determined in a pilot experiment.

## 3. Results

### 3.1. CCI-Induced Mechanical Hypersensitivity

**Figure 2:**
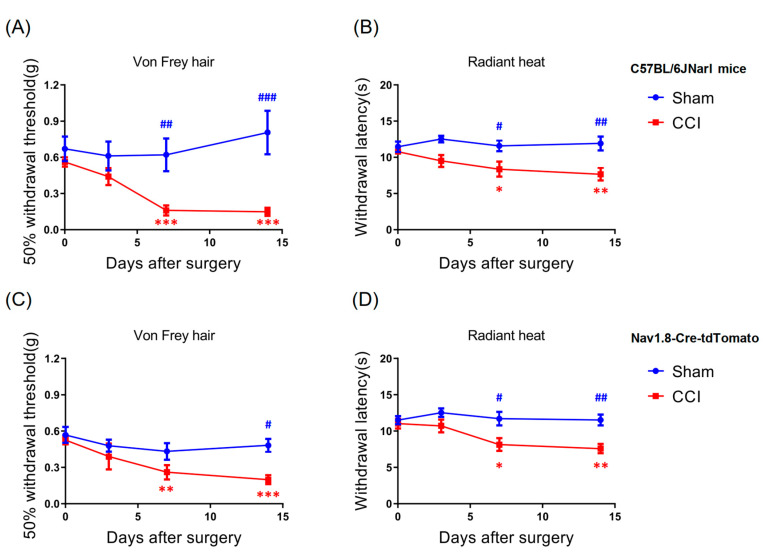
Effects of CCI-induced behavioral changes in wild-type mice and Nav1.8-tdTomato mice. (**A**,**B**) Both wild type (WT) and (**C**,**D**) Nav1.8-tdTomato mice developed mechanical hypersensitivity and thermal hyperalgesia after CCI surgery (WT-sham, *n* = 7; WT-CCI, *n* = 12; Nav-sham, *n* = 8; Nav-CCI, *n* = 15). * *p* < 0.05, ** *p* < 0.01 and *** *p* < 0.001, compared with the baseline of the CCI group; # *p* < 0.05, ## *p* < 0.01 and ### *p* < 0.001, compared with sham groups.

Previous studies have demonstrated that CCI induces mechanical hypersensitivity and thermal hyperalgesia in rodents [5,6]. In the present study, we first determined whether CCI effectively induced neuropathic pain behavior in the WT and Nav1.8-tdTomato mice. Before CCI, both mouse strains exhibited similar baseline thresholds of mechanical sensitivity (WT-sham, 0.671 ± 0.102, *n* = 7/7; WT-CCI, 0.562 ± 0.037, *n* = 12/12; Nav-sham, 0.568 ± 0.066, *n* = 8/8; Nav-CCI, 0.525 ± 0.036, *n* = 15/15; *p* > 0.05, two-way repeated measures ANOVA with Sidak post hoc test). Compared to baseline values, the 50% withdrawal threshold decreased significantly at 7 d after CCI (WT-CCI, t = 7.012, *p* < 0.001; *t* = 3.612, Nav-CCI, *p <* 0.01) and remained so for up to 14 d after CCI (WT-CCI, t = 7.223, *p* < 0.001; Nav-CCI, *t* = 4.46, *p <* 0.001) (Figure 2A,C). In addition, when compared to the sham group, the CCI group also showed a significant decline in mechanical threshold at 7 d (*t* = 3.816, *p* < 0.01) and 14 d after CCI (*t* = 5.449, *p* < 0.001) for the WT mice and at 14 d after CCI (*t* = 2.808, *p* < 0.05) for the Nav1.8-tdTomato mice (Figure 2A,C).

### 3.2. CCI-Induced Thermal Hyperalgesia

To determine whether CCI surgery also induced thermal hyperalgesia in the WT and Nav1.8-tdTomato mice, we examined heat-induced pain behavior at different timepoints after CCI surgery. Before the CCI, both groups of mice exhibited similar baseline thresholds of thermal sensitivity (WT-sham, 11.47 ± 0.723, *n* = 7/7; WT-CCI, 10.771 ± 0.358, *n* = 12/12; Nav-sham, 11.505 ± 0.563, *n* = 8/8; Nav-CCI, 11.029 ± 0.656, *n* = 15/15; *p* < 0.05, two-way repeated measures ANOVA with Sidak post hoc tests). Results revealed that, compared to baseline, the withdrawal latency decreased significantly at 7 d after CCI (WT-CCI, *t* = 2.95, *p* < 0.05; Nav-CCI, *t* = 2.888, *p <* 0.05; two-way repeated measures ANOVA with Sidak post hoc test), which was sustained for up to 14 d (WT-CCI, *t* = 3.804, *p* < 0.01; Nav-CCI, *t* = 3.455, *p <* 0.01) (Figure 2B,D). Moreover, both WT and Nav1.8-tdTomato mice showed a significant decline in thermal withdrawal latency compared with the sham group at 7 d (WT-CCI, *t* = 2.654, *p* < 0.05; Nav-CCI, *t* = 3.015, *p <* 0.05) and 14 d (WT-CCI, *t* = 3.525, *p* < 0.01; Nav-CCI, *t* = 3.332, *p <* 0.01) of CCI (Figure 2B,D). These results indicate that CCI caused peripheral nerve injury, which induced both mechanical hypersensitivity and thermal hyperalgesia in mice.

### 3.3. Mechanical Hypersensitivity Did Not Associate with Nerve-Injury Severity

**Figure 3:**
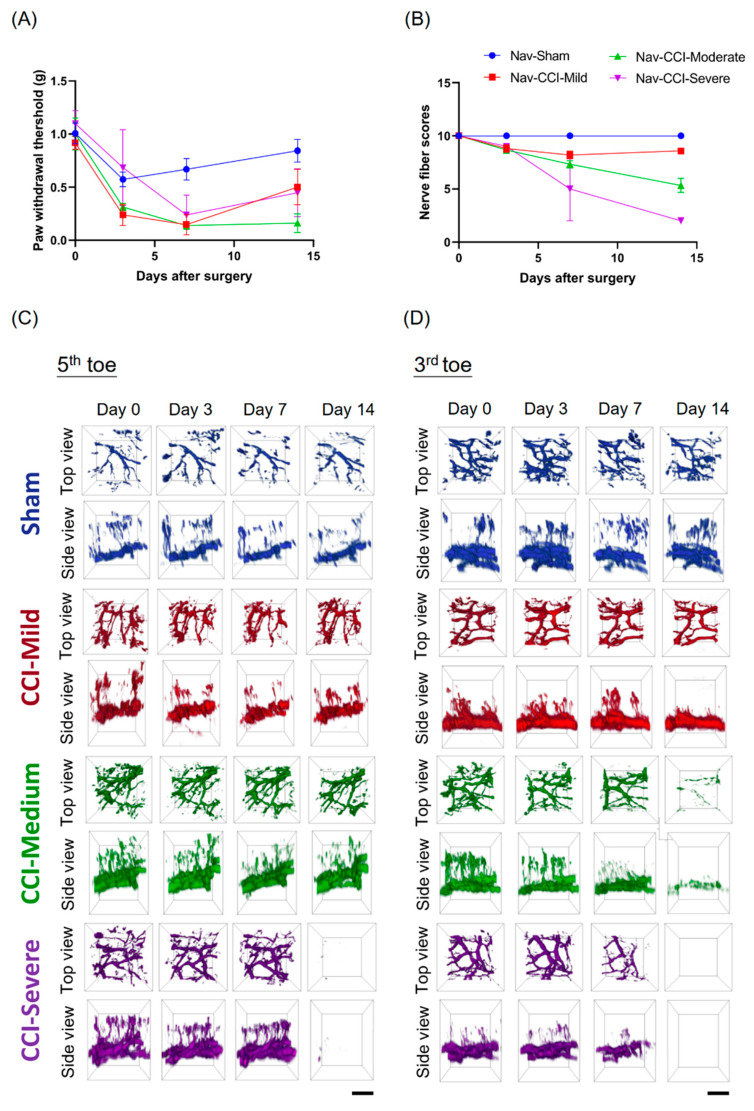
Association between nerve scores, intravital images and mechanical threshold. Mechanical hypersensitivity did not associate with nerve-injury severity. (**A**) The Nav1.8-tdTomato mice exhibited mechanical hypersensitivity and (**B**) the corresponding nerve scores after CCI surgery. The CCI-treated mice were divided into three subgroups according to nerve score: CCI-mild: 7–9; CCI-moderate: 3–6; and CCI-severe, 0–2. (**C**) Time-lapse observations of the fifth and (**D**) third toes in Nav1.8-tdTomato mice. Intravital microscope images showed that the mice had varying degrees of nerve degeneration in the nerve plexus and terminals, which corresponded with the severity of the CCI injury. Notably, the mice in the CCI-moderate group exhibited higher mechanical hypersensitivity than those in the CCI-mild and CCI-severe groups. Day 0 indicates baseline; scale bar: 50 μm.

We used intravital two-photon microscopy to investigate changes in the nerve plexus and nerve terminals during the development of CCI-induced neuropathy. Baseline values for the von Frey test, nerve-scoring test and intravital imaging, were all obtained before the surgery. The mice were subjected to the same tests at 3, 7 and 14 d after CCI surgery. The Nav1.8-tdTomato mice did exhibit mechanical hypersensitivity (Figure 3A) and the corresponding nerve scores (Figure 3B) after CCI surgery. The CCI-treated mice were then categorized into three subgroups, based on their nerve scores, as follows: CCI-mild, 7–9; CCI-moderate, 3–6; and CCI-severe, 0–2. Examples of co-registered intravital microscopic images of the Nav1.8-tdTomato nerve plexus in the tips of the third and fifth toes, captured on different days, are shown in Figure 3C,D. Our results showed that the level of CCI-injury severity was corresponded with the amount of degeneration in the nerve plexus and terminals in the Nav1.8-tdTomato mice. Notably, the moderately injured mice exhibited higher mechanical hypersensitivity than both the mildly and the severely injured mice. Combined, these results reveal that mechanical hypersensitivity was not associated with nerve-injury severity.

### 3.4. Degeneration of the Nerve Plexus and Terminals Was Associated with Nerve-Injury Severity

**Figure 4:**
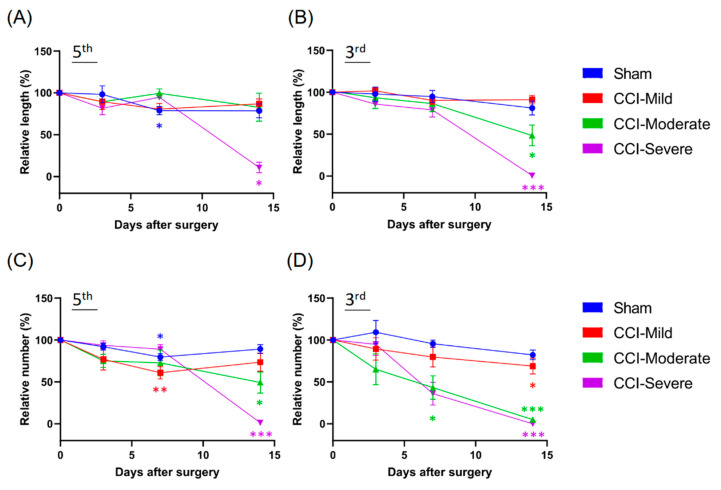
Changes in the sub-epidermal nerve plexus and nerve terminals in the third and fifth toe tips of mice in the CCI subgroups and the sham group. (**A**,**B**) Changes in the relative length of the nerve plexus. At 14 d after CCI surgery, the nerve plexus length was significantly reduced in both the third and the fifth toe tips of mice in the CCI-severe group. Mice in the CCI-moderate group only showed significant decreases in the length of the nerve plexus length in the third toe tip. (**C**,**D**) Changes in the relative number of nerve terminals. In the CCI-mild group, nerve terminals were significantly reduced in the fifth toe tip at 7 d after CCI but had recovered to baseline levels at 14 d. Both the CCI-moderate and the CCI-severe groups had significantly fewer terminals in the fifth toe tip at 14 d post CCI surgery. All CCI subgroups had significantly fewer terminals in the third toe tip at 14 d. * *p* < 0.05, ** *p* < 0.01 and *** *p* < 0.001, compared to baseline.

Changes in the nerve plexus and terminals were further analyzed and quantified after the reconstruction and co-registration of the intravital images. The CCI-injured mice were divided into CCI-mild (*n* = 5/5), CCI-moderate (*n* = 3/3) and CCI-severe (*n* = 1/2) subgroups, as described above. We compared the two indices that were derived from the level of degeneration in the nerve plexus and terminals of the tips of the third and fifth toes. Compared to baseline, the CCI-moderate mice showed significant decreases in the length of the nerve plexus in the third toe tip at 14 d post CCI (*q =* 6.161, *p* < 0.05, two-way ANOVA with Tukey’s post hoc test). In the CCI-severe group, the nerve plexus length was significantly reduced in both the third (*q =* 244.1, *p* < 0.001) and the fifth toe tip (*q =* 20.32, *p* < 0.05) (Figure 4A,B).

At 7 d after CCI, the number of nerve terminals in the CCI-mild mice was significantly reduced in the fifth toe tip (*q =* 7.509, *p* < 0.01, compared to baseline, two-way ANOVA with Tukey’s post hoc test) but had recovered to the baseline level (*q =* 3.445, *p* > 0.05) at 14 d. In addition, both CCI-moderate and CCI-severe mice had significantly fewer terminals in the fifth toe tip at 14 days after CCI surgery (CCI-moderate, *q =* 5.724, *p* < 0.05; CCI-severe, *q =* 100.4, *p* < 0.001). All three CCI subgroups had significantly fewer nerve terminals in the third toe tip at 14 d post CCI surgery (CCI-mild, *q =* 4.687, *p* < 0.05; CCI-moderate, *q =* 46.78, *p* < 0.001; CCI-severe, *q =* infinity, *p* < 0.001) (Figure 4C,D). These results suggested that the degeneration of the nerve plexus and terminals was associated with nerve-injury severity.

### 3.5. Mechanical Hypersensitivity Was Correlated with the Product of the Nerve Score and Number of Fibers Lost

**Figure 5:**
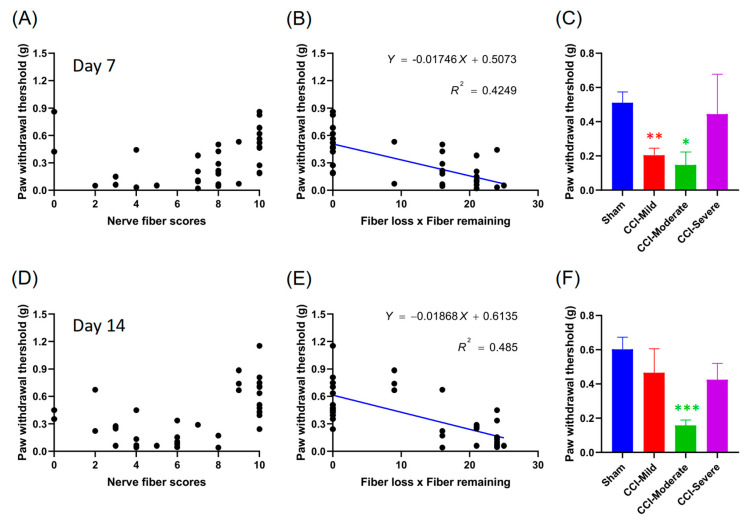
Correlation between mechanical threshold and the product of nerve scores. (**A**) Scatter plots showing data from individual mice data at 7 d after CCI surgery. (**B**) Mechanical hypersensitivity was correlated significantly with the product of the nerve score (surviving fibers) and the number of fibers lost (10−nerve score) at 7 d (*p* < 0.001). (**C**) The CCI-injured mice were divided into three subgroups according nerve score. Both the CCI-mild and the CCI-moderate groups showed mechanical hypersensitivity at 7 d. Panels (**D**–**F**) show data recorded at 14 d. Notably, only the CCI-moderate group exhibited mechanical hypersensitivity at 14 d, while the CCI-mild group had recovered and was not significantly different from the sham group. * *p* < 0.05, ** *p* < 0.01 and *** *p* < 0.001, compared with to the sham group.

To investigate the correlation between mechanical hypersensitivity and nerve score, we analyzed and presented data from individual mice as scatter plots and from the three subgroups as bar graphs, for 7 d (Figure 5A–C) and 14 d (Figure 5D–F) after CCI surgery. Mechanical hypersensitivity was reasonably well correlated with the product of the nerve score and number of fibers lost at 7 d (R^2^ = 0.4249, *p* < 0.001) (Figure 5B) and 14 d (R^2^ = 0.485, *p* < 0.001) (Figure 5E). However, only the CCI-moderate mice showed mechanical hypersensitivity at both 7 d (*q =* 4.654, *p* < 0.05 compared to sham, one-way ANOVA with Tukey’s post hoc test) (Figure 5C) and 14 d (*q =* 7.446, *p* < 0.001) (Figure 5F) after CCI surgery. The CCI-mild mice had recovered, with no significant differences from the sham group, at 14 d after CCI (*q =* 1.778, *p* > 0.05). These results suggest that the correlation between mechanical hypersensitivity and nerve score may be a useful estimation tool after the complete development of CCI-induced neuropathic pain.

### 3.6. Nerve Score at 7 d Post CCI Was Correlated with the Von Frey Test Threshold at 14 d

**Figure 6:**
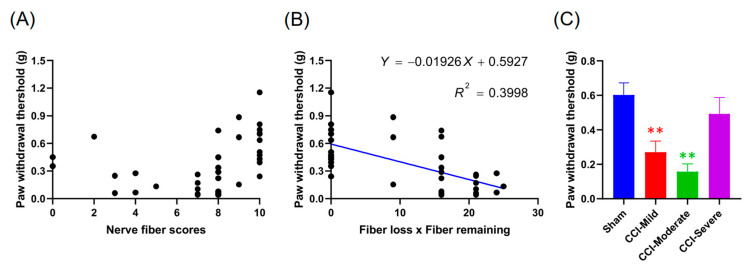
The estimative effect of the correlation between the product of the nerve score test at 7 d and the mechanical threshold at 14 d. (**A**,**B**) Scatter plots showed that nerve fiber condition at 7 d was significantly correlated with the mechanical threshold tested at 14 d (*p* < 0.001). (**C**) The CCI-injured mice were divided into three subgroups according to nerve score. Both the CCI-mild and the CCI-moderate groups but not the CCI-severe group, showed mechanical hypersensitivity. ** *p* < 0.01, compared to the sham group.

We tested whether early nerve fiber condition was a potential biomarker for the prognosis of mechanical hypersensitivity. The condition of the nerve fiber at 7 d was significantly correlated with the mechanical threshold tested at 14 d (R^2^ = 0.3998, *p* < 0.001) (Figure 6B). In addition, both the CCI-mild (*q =* 5.232; *p* < 0.01, compared with sham group, one-way ANOVA with Tukey’s post hoc test) and the CCI-moderate (*q =* 4.972, *p* < 0.01) but not the CCI-severe (*q =* 1.072, *p* > 0.05), mice exhibited mechanical hypersensitivity during using the nerve score expression at 7 d that was correlated with the mechanical threshold result at 14 d (Figure 6C). Combined, these data suggest the potential of using the whole-paw nerve-scoring approach to estimate pain-related responses during the early development of CCI pathology.

### 3.7. Quantification of Nerve Plexus and Terminals with Different Indices in Recategorized CCI Subgroups

**Figure 7:**
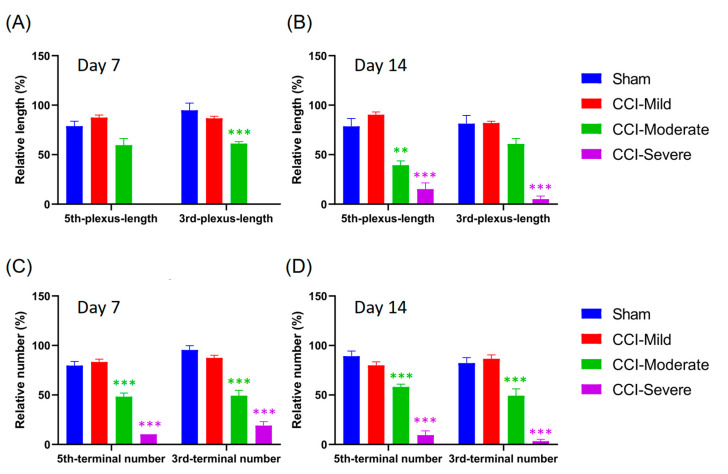
Small-ROI analysis of free nerve endings and sub-epidermal nerve plexus changes in the third and fifth toe tips of mice in the CCI subgroups and the sham group. The CCI-treated mice were recategorized into three subgroups according to the relative length and number of their remaining nerves: CCI-mild, 70–99%; CCI-moderate, 30–69%; and CCI-severe, 0–29%. (**A**,**B**) Changes in the relative length of the nerve plexus. The CCI-moderate group showed significantly reduced plexus lengths in the third and the fifth toe tips at 7 and 14 d, respectively. The CCI-severe group showed significantly reduced plexus lengths in the tips of both toes at 14 d after CCI surgery. (**C**,**D**) Both the CCI-moderate and the CCI-severe groups showed significantly reduced terminal numbers in the third and fifth toes tips at 7 and 14 d after CCI surgery. ** *p* < 0.01 and *** *p* < 0.001, compared to the sham group.

Although we had established that the whole-paw scoring approach could be used as a diagnostic and prognostic biomarker to assess neuropathic pain, it remained unknown whether using the indices, which were derived from a small volume selected from the intravital images, had the same effect. Hence, we recategorized the CCI-injured mice into three groups according to the relative length and number of nerves that remained after CCI surgery (CCI-mild, 70–99%; CCI-moderate, 30–69%; and CCI-severe, 0–29%). In this grouping, the CCI-moderate mice had significantly reduced plexus length in the third toe tip at 7 d (*q =* 6.951, *p* < 0.001 compared with the sham group, two-way ANOVA with Tukey’s post hoc test) (Figure 7A) and in the fifth toe tip at 14 d (*q =* 5.306, *p* < 0.01) (Figure 7B) post CCI. The CCI-severe group showed significantly reduced plexus length in the tips of both toes (third toe, *q =* 13.9, *p* < 0.001; fifth toe, *q =* 10.84, *p* < 0.001) at 14 d after CCI surgery (Figure 7B). In addition, both the CCI-moderate and the CCI-severe groups had significantly fewer nerve terminals in the tips of both toes at 7 d (CCI-moderate-third-terminal, *q =* 10.59, *p* < 0.001; CCI-severe-third-terminal, *q =* 19.53, *p* < 0.001; CCI-moderate-fifth-terminal, *q =* 8.339, *p* < 0.001; CCI-severe-fifth-terminal, *q =* 9.481, *p* < 0.001) and 14 d (CCI-moderate-third-terminal, *q =* 7.201, *p* < 0.001; CCI-severe-third-terminal, *q =* 18.84, *p* < 0.001; CCI-moderate-fifth-terminal, *q =* 7.236, *p* < 0.001; CCI-severe-fifth-terminal, *q =* 17.4, *p* < 0.001) after CCI surgery (Figure 7C,D).

### 3.8. Mechanical Hypersensitivity Was Associated with Indexing by Both the Relative Length and Number of Nerve Terminals Remaining After CCI

**Figure 8:**
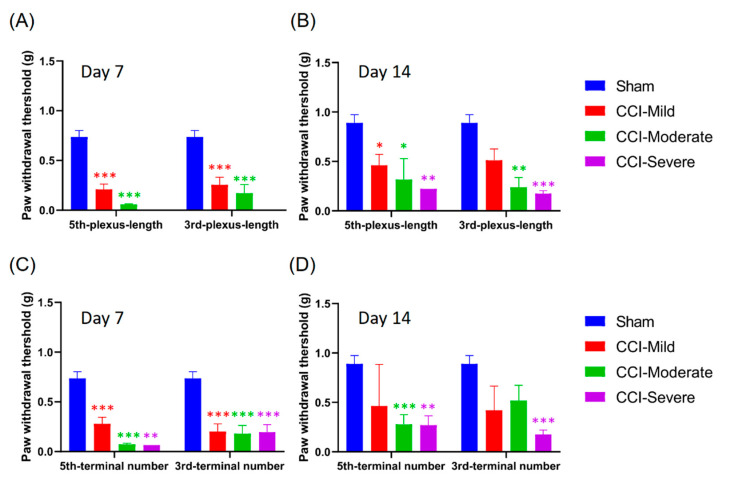
Association between mechanical threshold and the indices of the sub-epidermal nerve plexus and nerve terminals in the third and fifth toe tips. (**A**,**B**) Index of plexus length in the toe tips is associated with the mechanical threshold. The CCI-mild group showed mechanical hypersensitivity at 7 d when indexed by the third toe tip and at both 7 and 14 d when indexed by the fifth toe tip. The CCI-moderate group, indexed by either the third or the fifth toe tip, showed mechanical hypersensitivity at both 7 and 14 d after CCI surgery. The CCI-severe group, whether indexed by plexus length in the third or fifth toe tip, showed mechanical hypersensitivity only at 14 d. (**C**,**D**) The index of the number of nerve terminals in the toe tips is associated with mechanical threshold. The CCI-mild group showed mechanical hypersensitivity when indexed by number of terminals in the third or fifth toe at 7 d but not at 14 d. The CCI-moderate group indexed by the third toe showed mechanical hypersensitivity at 7 d, while the group indexed by the fifth toe showed hypersensitivity at both 7 and 14 d. The CCI-severe group, whether indexed by the third or the fifth toe, showed hypersensitivity at both 7 and 14 d. * *p* < 0.05, ** *p* < 0.01 and *** *p* < 0.001, compared to the sham group.

We compared the mechanical threshold in the subgroups that were categorized according the relative length of the nerve plexus after CCI surgery and examined whether the use of these indices was comparable to the whole-paw nerve-scoring approach. The CCI-mild mice that were indexed by plexus length in the third toe tip showed mechanical hypersensitivity at 7 d (*q =* 6.982, *p* < 0.001, compared with the sham group, two-way ANOVA with Tukey’s post hoc test), while those indexed according to the plexus in the fifth toe tip showed hypersensitivity at both 7 d (*q =* 8.803, *p* < 0.001) and 14 d (*q =* 4.396, *p* < 0.05). The CCI-moderate group indexed based on the index of plexus length in either the third (7 d, *q =* 6.712, *p* < 0.001; 14 d, *q =* 4.869, *p* < 0.01) or the fifth toe tip (7 d, *q =* 7.356, *p* < 0.001; 14 d, *q =* 4.291, *p* < 0.05) showed mechanical hypersensitivity at 7 and 14 d after CCI surgery. However, the CCI-severe group only showed hypersensitivity at 14 d, whether indexed by plexus length in the third (*q =* 6.243, *p* < 0.001) or the fifth toe tip (*q =* 4.995, *p* < 0.01) (Figure 8A,B).

Next, we investigated the mechanical threshold in subgroups categorized according to the number of nerve terminals remaining in the toe tips after CCI surgery. Mechanical hypersensitivity in the CCI-mild mice, whether indexed by the number of nerve terminals in their third (*q =* 7.896, *p* < 0.001) or fifth toe tips (*q =* 7.037, *p* < 0.001), was elevated at 7 d but not different from the sham group at 14 d (third toe, *q =* 3.056, *p* > 0.05; fifth toe, *q =* 2.772, *p* > 0.05). The CCI-moderate group, as indexed by nerve terminals in the third toe tip, showed hypersensitivity at 7 d (*q =* 6.871, *p* < 0.001), whereas that indexed by number of terminals in the fifth toe tip showed hypersensitivity at both 7 d (*q =* 9.52, *p* < 0.001) and 14 d (*q =* 6.02, *p* < 0.001). Mechanical sensitivity was significantly reduced in the CCI-severe group, whether indexed by number of terminals in the third or the fifth toe, both at 7 d (third toe, *q =* 7.452, *p* < 0.001; fifth toe, *q =* 4.95, *p* < 0.01) and 14 d (third toe, *q =* 7.002, *p* < 0.001; fifth toe, *q =* 5.7, *p* < 0.01) (Figure 8C,D). The CCI-mild and CCI-moderate groups were thus comparable to those obtained using the whole-paw nerve-scoring approach (Figure 5C,F).

### 3.9. Indexing by the Relative Length and Number of Nerves Remaining at 7 D Post CCI Was Associated with the Von Frey Test Threshold at 14 d

**Figure 9:**
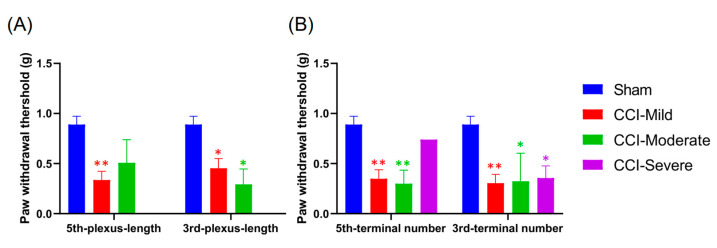
The ability of the indices of plexus length and number of terminals at 7 d to estimate mechanical threshold at 14 d. (**A**) The CCI-mild group showed mechanical hypersensitivity whether indexed by plexus length in the third or the fifth toe tip. The CCI-moderate group revealed mechanical hypersensitivity when indexed by plexus length in the third toe. (**B**) The CCI-mild and CCI-moderate groups displayed mechanical hypersensitivity whether indexed by number of nerve terminals in the third or the fifth toe tip. The CCI-severe group showed mechanical hypersensitivity when indexed by number of terminals in the third toe. * *p* < 0.05, ** *p* < 0.01, compared to the sham group.

We examined whether indexing mice by the relative length and number of nerves remaining at 7 d post CCI surgery estimated their mechanical threshold as effectively as the nerve-scoring approach. The CCI-mild group, whether indexed by plexus length in the third or the fifth toe tip, showed mechanical hypersensitivity (third toe tip: *q =* 4.178, *p* < 0.05, compared with sham group, two-way ANOVA with Tukey’s post hoc test; fifth toe tip: *q =* 5.588, *p* < 0.01). The CCI-moderate group exhibited mechanical hypersensitivity only when indexed by plexus length in the third toe tip (*q =* 4.67, *p* < 0.05) (Figure 9A). In addition, both the CCI-mild and the CCI-moderate group showed mechanical hypersensitivity when using the index of nerve terminals in the third toe tip (CCI-mild, *q =* 5.258, *p* < 0.01; CCI-moderate, *q =* 3.883, *p* < 0.05) or the fifth toe tip (CCI-mild, *q =* 5.089, *p* < 0.01; CCI-moderate, *q =* 5.154, *p* < 0.01). The CCI-severe group displayed mechanical hypersensitivity when indexed by nerve terminals in the third toe tip (*q =* 4.488, *p* < 0.05). These data indicated that using the indices derived from the relative changes in nerve plexus length and nerve terminal numbers estimated CCI-mild and CCI-moderate injury as effectively as the whole-paw nerve-scoring approach (Figure 6C).

## 4. Discussion

In this study, we found that CCI caused mechanical hypersensitivity and thermal hyperalgesia in both WT and Nav1.8-tdTomato mice. Using the nerve score derived from the whole hind paw, we found that mechanical hypersensitivity was significantly correlated with the product of injured and surviving nerve fibers but not with nerve injury alone. In addition, indices of nerve plexus length and terminal numbers, derived from a small volume of intravital images, similarly estimated the effect of CCI on the CCI-mild and CCI-moderate groups.

Nociceptive nerves innervate the skin and play a crucial role in the generation of neuropathic pain. The examination of sensory nerve terminals in the epidermis is a well-established approach that is used to estimate skin innervation and thereby investigate the integrity of nociceptive nerves in humans and animals [27,28,29]. Previous studies have shown that rats exhibited a significant loss in epidermal nerve fiber density and in thermal hyperalgesia at 14 days after CCI surgery [30]. In addition, the decrease in cutaneous nerve innervation and increase in neuropathic pain behavior (e.g., mechanical and thermal hypersensitivity) can be reversed by decompression in CCI-treated rats [21]. Other studies have also shown that CCI-treated animals exhibit significant mechanical hypersensitivity and thermal hyperalgesia at seven days after surgery [31]. Notably, CCI surgery has different effects on the different subpopulations of nerve terminals that are involved in cutaneous innervation. After initial loss, nonpeptidergic nerve fibers require a longer time to recover than peptidergic nociceptive fibers. In addition, eight weeks after CCI, ectopic sympathetic fibers were observed to have formed novel associations and were wrapped around sprouted peptidergic nociceptive fibers [32]. In summary, these studies indicate that CCI causes structural changes such as nerve injury, skin-sensory nerve degeneration and nerve sprouting. In addition, the pain behavior and phenotypes may be affected by the surviving nerve fibers and nerve terminals. Peripheral nerve injuries could induce plastic changes on the primary afferent fibers and on the spinal circuitry, both of which may relate to the emergence of neuropathic pain [33]. Further, primary somatosensory neurons in the DRG could be classified according to its specific anatomical and chemical attributes, including axonal conduction velocity, expression of different molecules and characteristic patterns of innervation of peripheral and central targets [34,35,36,37]. Previous studies have revealed that, following nerve injury, primary afferent nerves expressing Nav1.8 contribute to the abnormal conduction of sensory information, which facilitates repetitive firing in the DRG upon sensory stimulation [38,39,40]. Both electrophysiological and pharmacological results have demonstrated the indispensable role of Nav1.8 in multiple types of chronic pain states [41,42]. Genetically engineering the selective knockdown of the Nav1.8 protein prevented hyperalgesia and allodynia caused by either chronic nerve or tissue injury [43]. In addition, the amplitude of the compound action potentials increased in the myelinated and unmyelinated fibers of the ipsilateral sciatic nerve after sciatic nerve entrapment, which is similar to CCI. However, the proportion of Nav1.8-postivie DRG neurons co-expressed with different specific molecular markers, such as 50.6% of IB4 (nonpeptidergic C-nociceptors), 36.1% of substance P (SP) (peptidergic C-nociceptors) and 52.1% of CGRP (peptidergic C-nociceptors with some A-fiber afferents) [9]. Morphological studies revealed that CCI-induced significantly CGRP-positive and SP-positive IEFD loss but only the SP-positive cutaneous nerve fibers could regenerate after nerve decompression [21,44]. Moreover, CCI induced a two-fold enhancement of IB4-CGRP overlapping comparing with ipsilateral and contralateral side of spinal cord dorsal horn which could be reversed after nerve decompression [22]. Other study also showed that a transient decreased of IB4-positive labeling (in 1 week) in the spinal cord dorsal horn at 7 d but returned to basal levels 14 d after CCI surgery [33]. Combined, these results suggest that Nav1.8 plays a crucial role in the development and maintenance of neuropathic pain which may have specific sub-population involvement in the peripheral and central sensitization.

Peripheral nerve injury also triggers the local neurogenic inflammation which enhanced capillary permeability triggering by the activation of mast cells, with also recruitment of neutrophils and macrophages [45,46]. After nerve injury, the accumulation of macrophage, neutrophil and lymphocyte in the peripheral nervous system contribute to the peripheral sensitization which are mediated by several cytokines, such as tumor necrosis factor-α (TNFα), interleukin-1β (IL-1β), chemokine (C-C motif) ligand 2 and C-C chemokine receptor 2 [47]. In addition, cytokines may play a crucial role in the CCI-induced hyperalgesia with potential mechanism of inflammation in and around the nerve [48]. Report showed that CCI-induced elevation of IL-1β and IL-6 expression in sciatic nerve at 7 d and TNF at 14 d [49]. Interestingly, these enhanced pro-inflammatory cytokines (TNFα, IL-1β and IL-6) expression in DRG corresponded to the development of CCI-induced mechanical and thermal allodynia [50]. These data suggest that the degeneration changes after CCI observed in the present study may induce hypersensitivity through complicated means.

Intravital microscopy is a noninvasive approach that offers numerous advantages in real-time observation and permits repeated measurements over multiple time windows. This enabled us to investigate cutaneous nerve degeneration and the survival of sensory nerve terminals during the CCI pathological process. In addition, the use of intravital microscopy, in lieu of traditional staining of frozen tissue sections, reduced the number of animals required for the study. Recently, intravital microscopy was used to explore nerve degeneration and immune-cell movement in the ears of macrophage-specific transgenic mice [51]. Other studies have also reported the use of in vivo two-photon microscopy for longitudinal observation of dynamic changes in nerve endings under the skin of the paw [52,53]. To our knowledge, our study is the first to explain the lack of correlation between nerve-injury severity and neuropathic pain behavior. In addition, this noninvasive approach is a potential prognostic tool for future human and animal studies investigating cutaneous pathological features in various conditions involving complicated neuropathic pain.

The evaluation of IEFD via PGP9.5 immunostaining of skin-punch biopsies is one of the diagnostic tools available for neuropathy [27,54]. However, the severity of cutaneous IEFD depletion does not correlate with neuropathic pain [2,3,54]. An animal study that applied polyethylene cuffs of varying sizes on the sciatic nerve induced both nerve degeneration and neuropathic pain but did not find a correlation between pain behavior and the number of nerve fibers [3]. In addition, a human study revealed no significant correlation between pain intensity and IEFD in skin biopsies from the lower limb [2].

In this study, we found that moderately and severely injured mice had similar numbers of ATF-3^+^ neurons (Supplementary Figure S1). ATF-3 is a member of the activating transcription factor/cAMP-responsive element-binding protein family of transcription factors that are induced in a variety of tissues under stress [17]. In addition, ATF-3 has been found to be upregulated in the cell bodies of sensory and motor neurons [18,55,56]. However, several groups have reported no correlation between ATF-3 expression and pain behavior in various models of nerve injury [18,57,58]. Hence, ATF-3 expression cannot be used as a biomarker for neuropathic pain.

The present study has several limitations. Intravital microscopy of the Nav1.8-positive nerve plexuses and terminals was captured at 0, 3, 7 and 14 days after CCI surgery. The CCI-injured mice were then divided into three subgroups for further analysis, based on either the whole-paw nerve-scoring approach or indices derived from quantification of the relative change in nerve plexus length and number of terminals. However, the size of the CCI-severe group obtained from the indexing derived from the quantification of intravital images was smaller (*n* = 1) than that of other groups. Nevertheless, using the nerve-scoring approach and the indices of relative change in the nerve plexus and terminals to estimate mechanical threshold yielded similar results for the CCI-mild and CCI-moderate groups. Whether all three levels of CCI injury as classified according to the different approaches would show the same phenotype or display varying effects of time and group, needs further investigation.

## 5. Conclusions

In summary, we demonstrated that two methods of assessment—whole-paw nerve scoring using an index combining surviving nerves and injured nerves and indices derived from the quantification of relative changes in nerve plexus and terminals—were correlated with evoked mechanical hypersensitivity. This finding implies that both surviving and injured nerves are necessary to initiate evoked neuropathic pain. In addition, both of these methods may have the estimative effect to be used as a diagnostic and prognostic biomarker for neuropathic pain.

## Supplementary Materials

The following are available online at https://www.mdpi.com/2073-4409/9/11/2414/s1, Figure S1: CCI affected similar numbers of AMP-dependent transcription factor-3-positive neurons in moderately and severely injured mice.
