# Supplementary material for: An Index Combining Lost and Remaining Nerve Fibers Correlates with Pain Hypersensitivity in Mice
Source: Cells. 2020 Nov 4;9(11):2414. doi: 10.3390/cells9112414 (PMC7694241; doi:10.3390/cells9112414)
Supplement: Supplementary file 1 [file cells-09-02414-s001.zip › Supporting information/cells-983750 - Supporting information.pdf]

# Supporting information

## CCI affected similar numbers of AMP-dependent transcription factor-3-positive neurons in moderately and severely injured mice

We examined neuropathic injury in the DRG of mice at 10 d post CCI surgery by staining for cyclic AMP-dependent transcription factor-3 (ATF-3), a well-known neuropathic pain marker [46]. The results reveal a similar number of ATF3+ L4 DRG cells in a moderately (CCI #2540) and a severely injured mouse (CCI #2996) (Figure S1). Therefore, the classical injury-related immunostaining approach did not provide adequate information for determining the severity of nerve terminal loss.

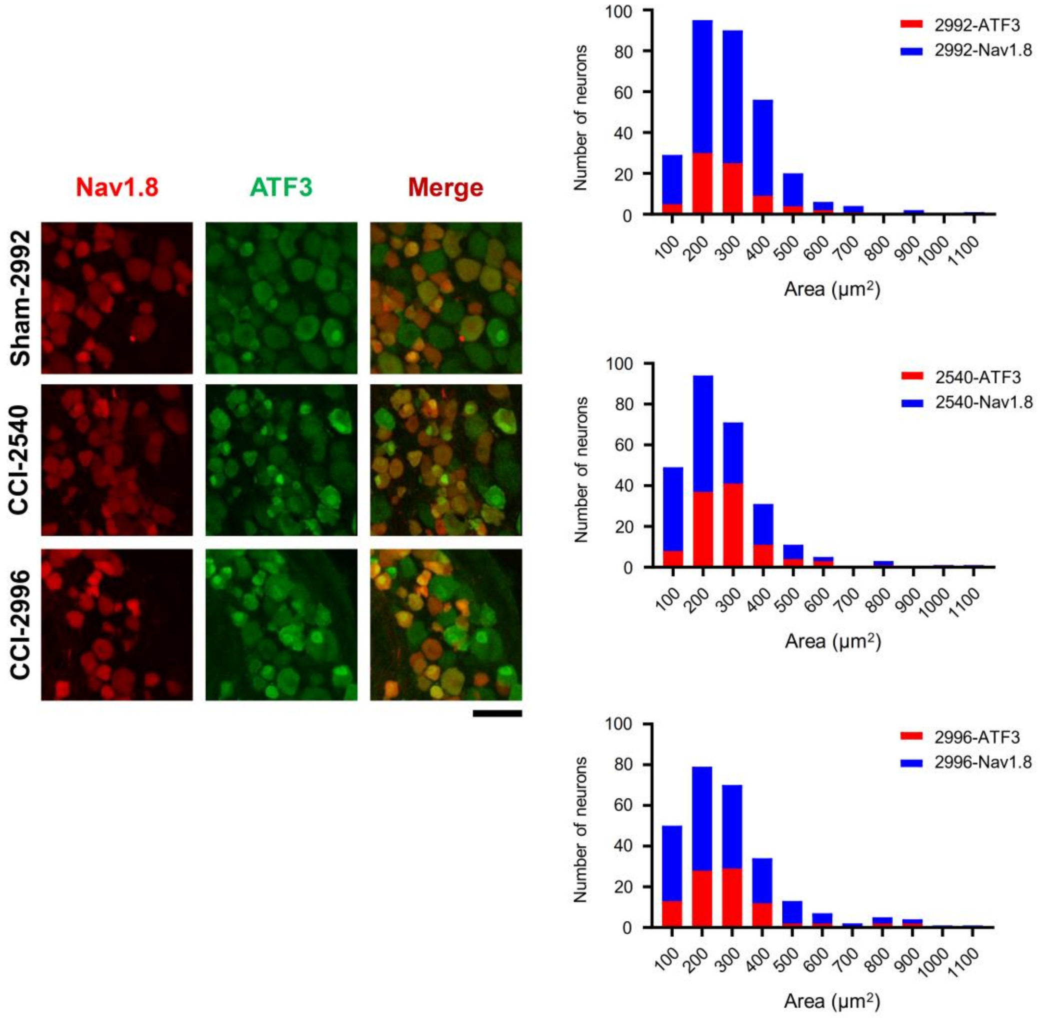

**Figure 1. Classification of ATF-3+ DRG cell size by the degree of injury in CCI-treated mice.** A moderately injured mouse (CCI #2540) had a similar number of ATF3+ L4 DRG cells to a severely injured mouse (CCI #2996). Scale bar: 60 μm.
