# Supplementary figures and images for: An Index Combining Lost and Remaining Nerve Fibers Correlates with Pain Hypersensitivity in Mice
Source: Cells. 2020 Nov 4;9(11):2414. doi: 10.3390/cells9112414 (PMC7694241; doi:10.3390/cells9112414)

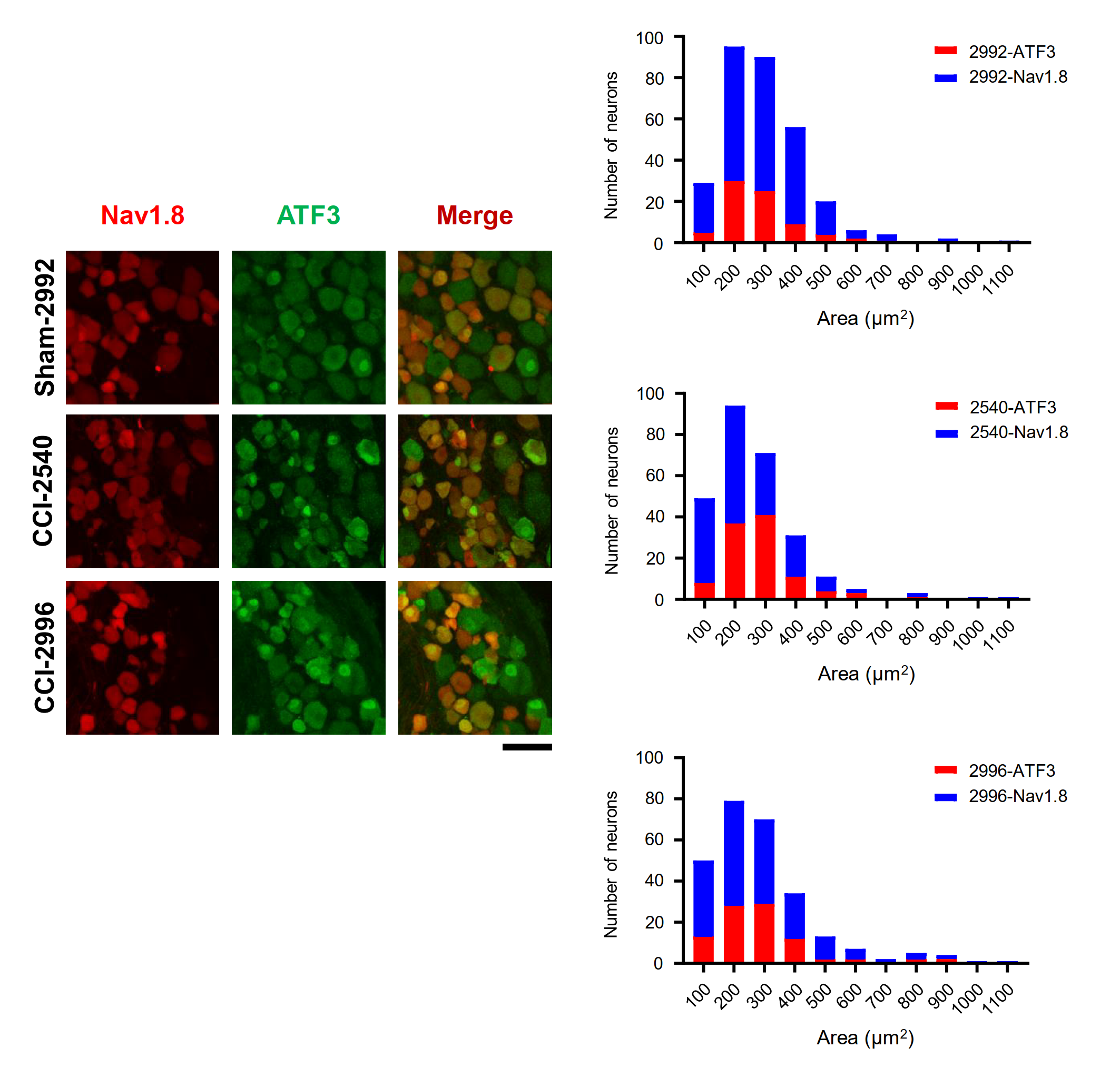

Supplement: Supplementary file 1 [file cells-09-02414-s001.zip › Supporting information/Fig_S1.tif]
